# Corrosion Inhibition of Mild Steel and 304 Stainless Steel in 1 M Hydrochloric Acid Solution by Tea Tree Extract and Its Main Constituents

**DOI:** 10.3390/ma14175016

**Published:** 2021-09-02

**Authors:** Jae-Yeon Kim, Inji Shin, Jai-Won Byeon

**Affiliations:** 1 Program of Material Science and Engineering, Convergence Institute of Biomedical Engineering and Biomaterials, Seoul National University of Science and Technology, Seoul 01811, Korea; jykim0517@seoultech.ac.kr or; 2 Department of Mining and Geological Engineering, University of Arizona, Tucson, AZ 85721, USA; 3 Department of Fine Chemistry, Seoul National University of Science and Technology, Seoul 01811, Korea; 4 Department of Materials Science and Engineering, Seoul National University of Science and Technology, Seoul 01811, Korea

**Keywords:** steel, corrosion inhibition, tea tree extract, raman spectroscopy

## Abstract

Tea tree extract, containing antioxidant constituents α-terpineol, terpinen-4-ol, and α-terpinene, has a wide range of applications in the cosmetic, food, and pharmaceutical industries. In this study, tea tree extract showed an anticorrosive effect under 1 M HCl solution on mild steel (MS) and 304 stainless steel (STS). Uniform corrosion for MS and pitting corrosion for STS at 298 K were retarded, with inhibition efficiencies of 77% and 86%, respectively. The inhibition of uniform and pitting corrosion was confirmed by scanning electron microscopy and laser scanning confocal microscopy in terms of surface roughness and pitting morphologies. The most effective constituent contributing to the inhibitory performance of tea tree extract was revealed to be α-terpineol, with an inhibition efficiency of 83%. The adsorption of tea tree extract was confirmed by surface characterization analysis using Fourier transform infrared spectroscopy, Raman spectroscopy, and Electrochemical impedance spectroscopy. Interestingly, G- and D-peaks of Raman spectra were detected from the inhibited steels, and this finding is the first example in the corrosion inhibition field. The anticorrosion mechanism can be explained by the formation of organic-Fe complexes on the corroded steel surface via electron donor and acceptor interactions in the presence of an oxygen atom of the hydroxyl group or ether of organic inhibitors.

## 1. Introduction

Organic inhibitors containing heteroatoms, such as oxygen, nitrogen, sulfur, and phosphorus atoms easily form a layer of organic-iron complexes by absorption on steel surfaces [1,2,3,4,5,6,7]. The adsorption layer acts as a physical barrier between the steel surfaces and the corrosive environment to provide effective protection against corrosion [1,2,3,4,5]. This phenomenon has been applied to restrain the internal corrosion of steel pipelines and the unexpected dissolution of steel structures during the acidizing process; therefore, the corrosion life of steel can be extended by a corrosion inhibitor [6].

Organic materials extracted from natural plants have been of great interest as promising alternatives to chemically synthesized compounds because they are eco-friendly, harmless to humans, and cheaper than synthetic compounds [8,9]. A variety of plant extracts have been reported as effective corrosion inhibitors in acid solutions (e.g., hydrochloric acid and sulfuric acid), particularly for mild steel [8,9,10,11,12,13,14,15,16]. Revealing the phytochemical constituents responsible for the corrosion inhibition is very important in order to clearly explain the inhibitory performance and mechanism of plant extracts. A few studies have been reported on the effective constituents of plant extracts, including ascorbic acid in orange peel extract [10], lawsone in henna extract [11], ellagic acid in punica granatum peel [12], arbutin of asteraceae extract [13], *N*-methyllaurotetanine in cryptocarya nigra extract [14], geissospermine of geissospermum leaf extract [15], and lutein in marigold flower extract [16]. In general, the inhibition mechanism based on effective constituents has been discussed through thermodynamical deduction and/or computational approaches, and there is a lack of studies reporting analytical evidence about the layer of organic-iron complexes.

In this study, the inhibitory action of tea tree extract on steel corrosion was investigated in hydrochloric acid solution. Inhibitory performance was tested on non-passivated mild steel (MS) exhibiting uniform corrosion and passivated 304 stainless steel (STS) exhibiting pitting corrosion. Particularly, tea tree extract has a corrosion inhibitory effect that has never been reported, and it was selected as a green organic inhibitor because of its availability, economical price, and good antioxidant properties [17,18,19]. In this work, the effective constituents contributing to the inhibition performance of tea tree extract were investigated, and its corrosion inhibitory mechanism was discussed based on the results of surface characterization using (Fourier transform infrared) FTIR and Raman spectroscopy (DXRTM 2 Raman Microscope, Thermo Fisher Scientific, Waltham, MA, USA).

## 2. Materials and Methods

### 2.1. Weight Loss Measurement

Nominal compositions of MS and STS used in this study are presented in Table 1. The specimens with 20 × 20 × 5 mm dimensions were mechanically ground using different grades of SiC paper (from 220 to 2000), rinsed with distilled water in an ultrasonic cleaning bath, and dried in the oven. These specimens were weighted prior to use, then immersed in a solution of 1 M HCl at 298, 308, 323, and 333 K. MS specimens and STS specimens were immersed for 24 and 168 h, respectively. The water-soluble tea tree extract was added to a 500 mL electrolyte at concentrations ranging from 0.15 to 2.25 g/L. The specimens retrieved from the test solution were washed with distilled water, dried in the oven, and subsequently reweighted up to a 1 mg unit. The corrosion rate was calculated using Equation (1):

(1) $$\mathrm{Corrosion}\;\mathrm{rate}\;\left(\mathrm{mg}/{\mathrm{cm}}^{2}\cdot h\right)=\frac{∆m}{A\;\times \;T}$$

where Δm is the weight loss of coupons (unit: mg), A is the exposure area (unit: cm^2^), and T is the immersion time (unit: h).

The inhibition efficiency (IE) was calculated from the weight loss results using Equation (2):

(2) $$\mathrm{Inhibition}\;\mathrm{efficiency}\;\left({\mathrm{IE}}_{\mathrm{WL}},\;\%\right)=\frac{{\mathrm{CR}}_{0}-{\;\mathrm{CR}}_{\mathrm{inh}}}{{\mathrm{CR}}_{0}}\times 100,$$

where CR_inh_ and CR_0_ are the corrosion rate in the presence and the absence of the corrosion inhibitor, respectively. Surface morphology of the retrieved specimens was observed by scanning electron microscope (SEM) (JSM-6700A, JEOL LTD., Tokyo, Japan) and laser scanning confocal microscopy (LSCM), (LSM 800 MAT, Carl Zeiss Co. Ltd., Oberkochen, Germany).

### 2.2. Gas Chromatography–Mass Spectrometry

Constituents of tea tree extract were analyzed by gas chromatography–mass spectrometry (GC-MS) (Hewlett-Packard Co., Palo Alto, CA, USA) using HP 6890 (gas chromatograph) and HP 5973 (mass spectrometer) models. About 1 μL of tea tree extract sonicated with hexane was used, and detailed information of GC–MS analysis is summarized in Table 2. α-Terpineol (96% purity, Alfa Aesar, Ward Hill, MA, USA), 1,8-cineole (99% purity, Alfa Aesar, Ward Hill, MA, USA), terpinen-4-ol (≥95% purity, Sigma Aldrich, Burlington, MA, USA), α–terpinene (≥95% purity, Sigma Aldrich, Burlington, MA, USA), and γ–terpinene (97% purity, Sigma Aldrich, Burlington, MA, USA) were analyzed as reference materials to determine the constituents. These organic materials have been known for the main phytochemical constituents of tea tree extract [20].

### 2.3. Electrochemical Measurements

The specimens used in the electrochemical experiments were embedded in epoxy resin, except for 1 cm^2^ of the surface exposed to the electrolyte. The exposed surface was mechanically ground in the same manner as described in Section 2.1. After 30 min of immersion at open circuit potential (OCP) in 1 M HCl solution, polarization measurements were taken, and electrochemical impedance spectroscopy (EIS) was performed using a potentiostat (VersaSTAT 4, Princeton Applied Research Co. Ltd., Oak Ridge, TN, USA). The three-electrode cell consisted of the saturated calomel reference electrode (SCE), Pt wire counter electrode, and working electrode of the steel specimens. Polarization curves were obtained at a scan rate of 1 mV/s from −0.65 to −0.10 V. The IE was calculated from the polarization curves using Equation (3):

(3) $$\mathrm{Inhibition}\;\mathrm{efficiency}\;\left({\mathrm{IE}}_{\mathrm{PD}},\;\%\right)=\frac{I_{0}-{\;I}_{\mathrm{inh}}}{I_{0}}\times 100,$$

where I_inh_ and I_0_ are the corrosion current densities of the specimen in the presence and absence of the corrosion inhibitor, respectively. The I_inh_ and I_0_ were derived through the Tafel extrapolation method of the polarization curves using DC data analysis software (IVMAN, WonATech Co., Ltd., Seoul, Korea).

EIS was performed at OCP in a frequency range from 0.1 to 10 MHz with a signal of amplitude of 10 mV. Commercial software (ZSimpwin, AMETEK Scientific Instrument, Berwyn, Pennsylvania, USA) was used for the fitting of Nyquist plots. The IE was calculated with R_CT_ values of the EIS results using Equation (4):

(4) $$\mathrm{Inhibition}\;\mathrm{efficiency}\;\left({\mathrm{IE}}_{\mathrm{EIS}},\;\%\right)=\frac{R_{\mathrm{CT}}^{\mathrm{inh}}-{\;R}_{\mathrm{CT}}^{0}}{R_{\mathrm{CT}}^{\mathrm{inh}}}\times 100,$$

where $R_{\mathrm{CT}}^{\mathrm{inh}}$ and $R_{\mathrm{CT}}^{0}$ are the charge transfer resistance values in the presence and absence of the corrosion inhibitor, respectively.

### 2.4. FTIR and Raman Spectroscopy

The corroded surface of steel specimens was characterized using (FTIR) spectroscopy (Nicolet iS50, Thermo Fisher Scientific, USA) and Raman spectroscopy. The surface of the specimens was polished until a mirror-like surface was obtained using SiC paper and 1 μm of Al_2_O_3_ paste, rinsed with distilled water in an ultrasonic cleaning bath, and then dried in the oven. The prepared specimens were immersed in 1 M HCl solution in the presence or absence of the inhibitor at 298 K. The specimens retrieved from the test solution were carefully washed with distilled water and dried in the oven at 40 ℃ for 1 h. Raman spectra were obtained using a 10 mW power and 532 nm wavelength laser as an excitation source. In order to prevent thermal degradations (e.g., phase transformation of Fe oxides or burning of organic materials), the laser power used in this study was optimized, as shown in the Supporting Information (Figure S1). The FTIR and Raman spectra of tea tree extract and its constituents (i.e., α-terpineol (96% purity, Alfa Aesar, Ward Hill, MA, USA), 1,8-cineole (99% purity, Alfa Aesar, Ward Hill, MA, USA) and terpinen-4-ol (≥95% purity, Sigma Aldrich, Burlington, MA, USA)) were obtained by analyzing their raw materials.

## 3. Results and Discussion

### 3.1. Weight Loss Measurements

**Figure 1:**
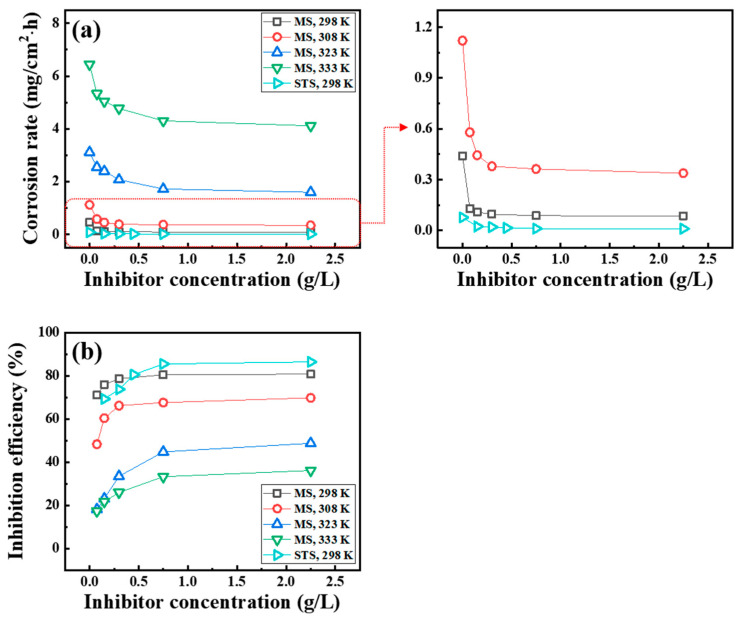
Variation in (**a**) corrosion rate and (**b**) the IE_WL_ for MS and STS specimens obtained from the weight loss test in 1 M HCl solution.

The corrosion rate (Figure 1a) and IE_WL_ (Figure 1b) of MS and STS were measured in 1 M HCl solution at various temperatures. The corrosion rate of MS and STS specimens at room temperature decreases to 77% and 86%, respectively, with the addition of 0.75 g/L of tea tree extract. As the temperature increases, the IE_WL_ decreases from 77% at 298 K to 36% at 333 K. In the presence of tea tree extract, the corrosion of MS and STS is inhibited by the adsorption phenomenon. The decrease in IE_WL_ at the raised temperature may be attributed to the desorption of tea tree extract from the steel surface and/or the increase in the reaction activity of corrosive ions on the unprotected surface [21,22].

**Figure 2:**
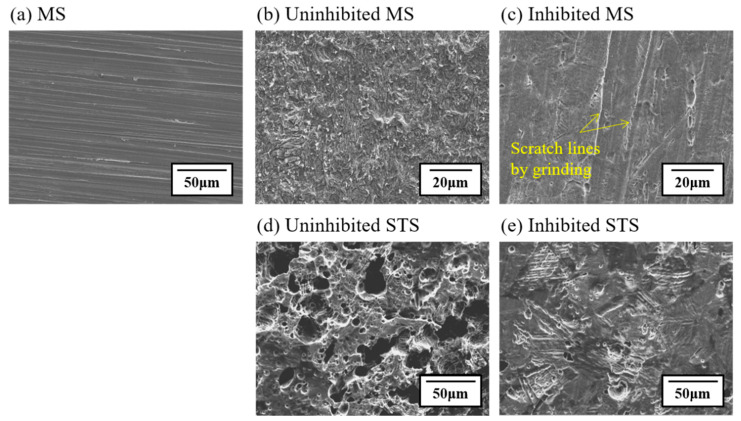
SEM micrographs for the corroded specimens immersed in 1 M HCl solution at 298 K containing 0.75 g/L of tea tree extract; (**a**) ground MS specimen without any treatment; (**b**) uninhibited MS; (**c**) inhibited MS; (**d**) uninhibited STS; (**e**) inhibited STS.

Figure 2 shows SEM micrographs of the surface of MS (Figure 2a–c) and STS (Figure 2d,e) specimens after immersion in 1 M HCl solution at 298 K in the absence and presence of 0.75 g/L of tea tree extract. These SEM micrographs were observed in the morphological analysis of the corroded surface. Figure 2a shows the surface morphology of the ground MS specimen without any treatment. Both uninhibited (Figure 2b) and inhibited (Figure 2c) MS specimens exhibit a uniform corrosion morphology. The surface of the uninhibited specimen is rough due to severe corrosion damage in 1 M HCl solution, while the surface of the inhibited specimen exhibits a relatively smoother morphology than that of the uninhibited one. Interestingly, some parts of the inhibited specimen remain intact even in acid solution due to the inhibitory role of tea tree extract; therefore, the morphology was comparable to the initial state (Figure 2a,c). A typical pitting corrosion morphology was observed in both uninhibited (Figure 2d) and inhibited (Figure 2e) STS specimens. Particularly, the size and number of pits were reduced by the addition of the inhibitor, tea tree extract.

**Figure 3:**
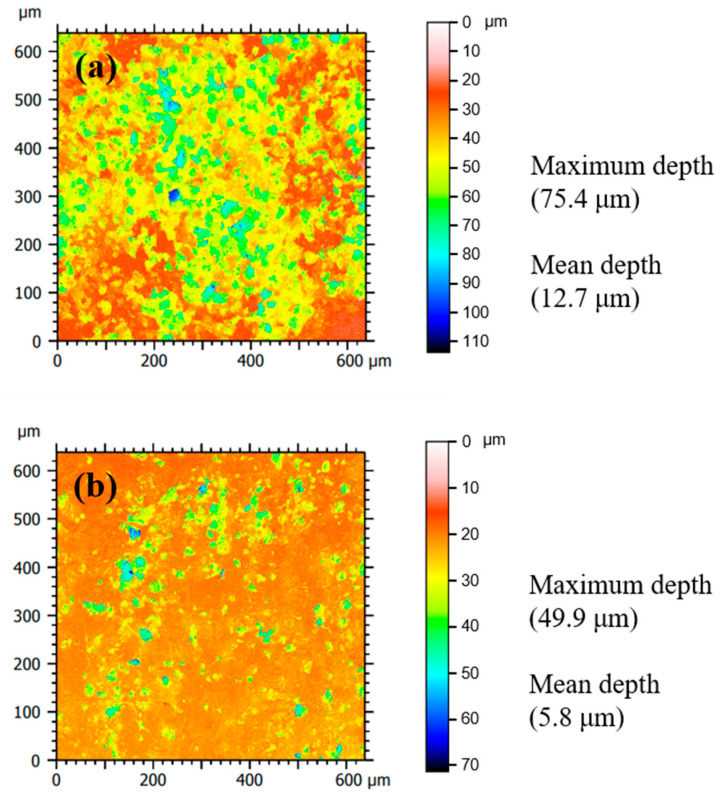
LSCM images showing the surface morphology of corroded STS specimens after immersion in 1 M HCl solution at 298 K: (**a**) uninhibited and (**b**) the inhibited specimens by 0.75 g/L of tea tree extract.

The characteristics of pits for STS specimens were quantitatively analyzed using LSCM (Figure 3). The STS speciemens were immersed in 1 M HCl solution at 298 K in the absence (Figure 3a) and presence (Figure 3b) of the tea tree extract of 0.75 g/L. Color scales next to LSCM images indicate the depth of the pit in micrometers. Analysis of mean depth and maximum depth from LSCM images was conducted using MountainLab^®^ software. In order to guarantee the reproducibility of characterization results, additional LSCM images were obtained at different points for the uninhibited and inhibited specimens, as shown in the Supporting Information (Figures S2 and S3). The pitting corrosion area (i.e., blue color area), the mean depth of pits (from 12.7 to 5.8 μm), and the maximum pit depth (from 75.4 to 49.9 μm) for the inhibited STS specimen decreased compared to the uninhibited one. These SEM and LSCM results indicate that the uniform corrosion for MS and the pitting corrosion for STS were effectively protected in 1 M HCl solution with the addition of tea tree extract.

### 3.2. Polarization Measurements

**Figure 4:**
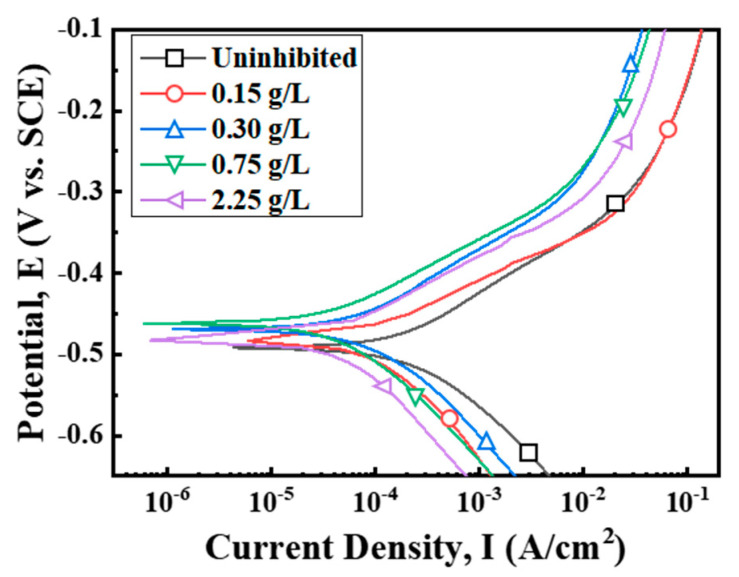
Polarization curves for mild steel specimen in 1 M HCl at 298 K with different concentrations of tea tree extract.

The effect of tea tree extract on the anodic and cathodic polarization behavior of MS was studied by electrochemical polarization measurements (Figure 4). The electrochemical parameters derived by Tafel extrapolation are presented in Table 3. The corrosion potentials (E_corr_) for the inhibited conditions slightly shifted to the anodic direction within about 30 mV compared with the uninhibited one. Organic corrosion inhibitors can be classified according to the differences in E_corr_ values, at least 85 mV positively or negatively, compared to the value of the uninhibited condition. The difference in E_corr_ values within 85 mV indicates a mixed-type inhibitor, which simultaneously delays hydrogen evolution in the cathodic site and steel dissolution in the anodic site [23]. Therefore, tea tree extract might be a mixed-type inhibitor. This explanation is confirmed from the displacements of both anodic and cathodic branches towards lower current density with the addition of tea tree extract. Corrosion current density (I_corr_) for the inhibited MS specimen could be reduced to about 80% compared with that of the uninhibited one due to the inhibition of anodic and cathodic reactions.

### 3.3. Corrosion Inhibition Effect of Constituents

**Figure 5:**
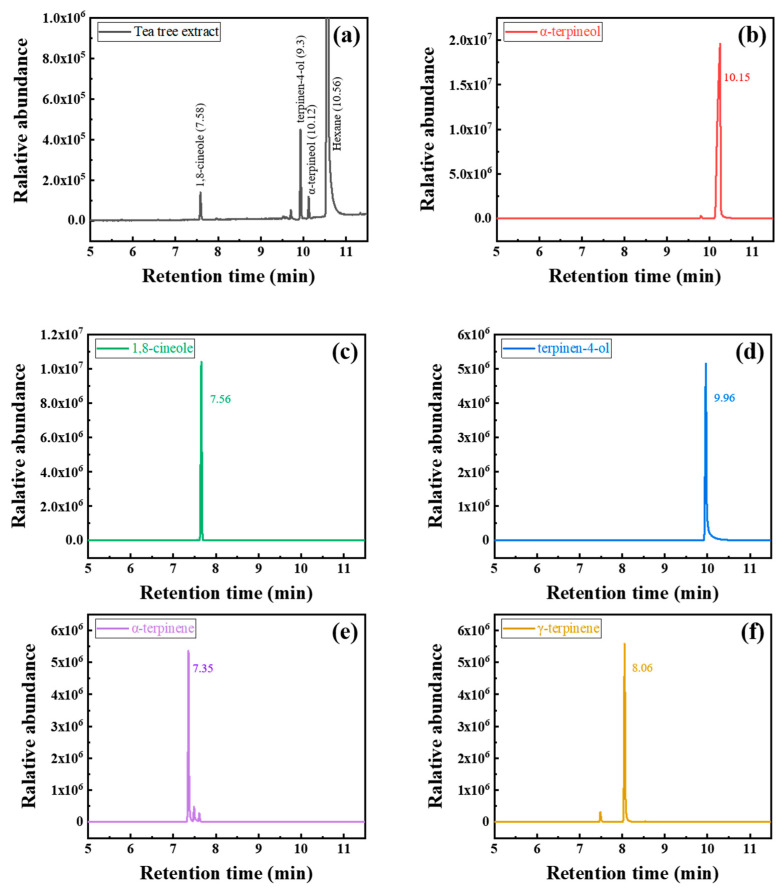
Chromatograms of (**a**) tea tree extract, (**b**) α-terpineol (**c**) 1,8-cineole, (**d**) terpinen-4-ol, (**e**) α–terpinene, and (**f**) γ-terpinene.

The organic constituents of tea tree extract were analyzed by GC–-MS, as shown in Figure 5. The water-soluble tea tree extract mainly consisted of α-terpineol, 1,8-cineole, and terpinen-4-ol.

**Figure 6:**
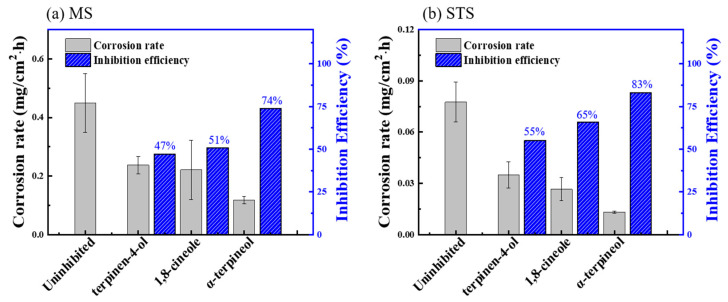
Corrosion rate and IE_WL_ for (**a**) MS and (**b**) STS specimens immersed in the 1 M HCl solution at 298 K containing 0.75 g/L of constituents.

In order to identify the active phytochemical constituents responsible for the inhibitive performance of tea tree extract, a weight loss test was carried out in 1 M HCl solution at 298 K, in which α-terpineol, 1,8-cineole, and terpinen-4-ol were added. The corrosion rate and IE_WL_ for MS (Figure 6a) and STS (Figure 6b) specimens are presented in Figure 6. The inhibitive performance of the constituents for both specimens increased in the following order: α-terpineol ≈ tea tree extract > 1,8-cineole > terpinen-4-ol. This result indicates that α-terpineol is the most effective constituent of tea tree extract on steel corrosion inhibition.

### 3.4. Surface Characterization

Tea tree extract successfully inhibited the corrosion of MS and STS specimens in 1 M HCl solution, as follows from the results of the weight loss test, electrochemical polarization measurement, SEM, and LSCM (Section 3.1 and Section 3.2). The corrosion inhibition behavior of organic materials is attributed to the protective layer formed by its adsorption phenomenon on the steel surface in corrosive solution [7,13,23,24]. This layer prevents the interaction between the corrosive ions (i.e., chlorides ions) and the steel surface by acting as the physical barrier. The surface characteristics of the inhibited specimens were analyzed by FTIR, Raman spectroscopy, and EIS with the aim to reveal the adsorption of tea tree extract.

#### 3.4.1. FTIR

**Figure 7:**
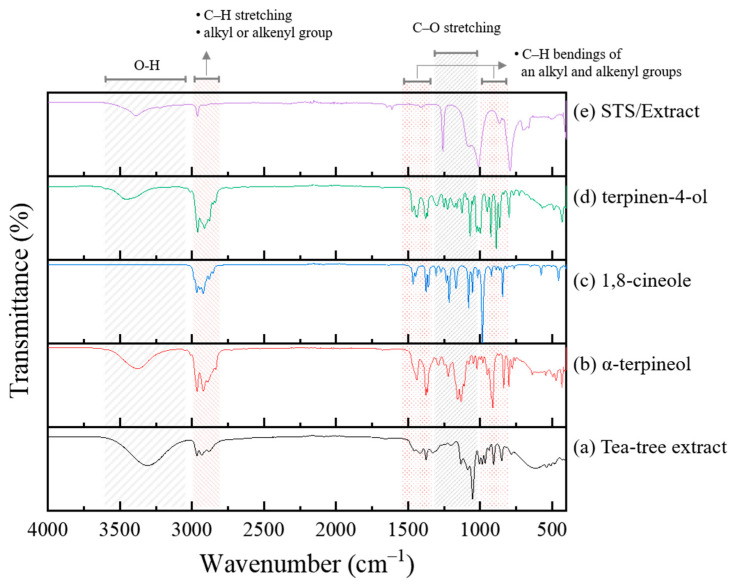
FTIR spectra of (**a**) tea tree extract, (**b**) α-terpineol, (**c**) 1,8-cineole, (**d**) terpinen-4-ol, and (**e**) the inhibited STS specimen in 1 M HCl solution containing 0.75 g/L of tea tree extract.

The main constituents of tea tree extract, α-terpineol, 1,8-cineole and terpinen-4-ol, were observed by FTIR (Figure 7a–d). A broad peak around 3300 cm^−1^ observed in α-terpineol and terpinen-4-ol indicates the O–H bond stretching vibration [13,24,25]. The C–H stretching of an alkyl or alkenyl group gives an absorption in the 2800–3000 cm^−1^ range [20,26]. The C–H bendings of an alkyl group and an alkenyl group occur in the 1300–1500 cm^−1^ [24,25,26,27] and 700–900 cm^−1^ ranges [7,26], respectively. Absorption peaks in the 1000–1300 cm^−1^ range are associated with the C–O stretching vibrations [7,13,26,27].

The FTIR spectrum was analyzed from the surface of the STS specimen after the immersion test in 1 M HCl solution containing tea tree extract (Figure 7e). The relative intensity and position of carbon peaks for the inhibited specimens were changed compared to those for the original tea tree extract and constituents. A notable change in Figure 7e was the weakening of relative intensity for the O–H vibration peak compared to the original tea tree extract and other constituents (Figure 5a,b,d). The constituents of tea tree extract such as α-terpineol and terpinen-4-ol are believed to be adsorbed through chemical interactions between their hydroxyl (O–H) group and the steel surface [7,13,24].

#### 3.4.2. Raman Spectroscopy

**Figure 8:**
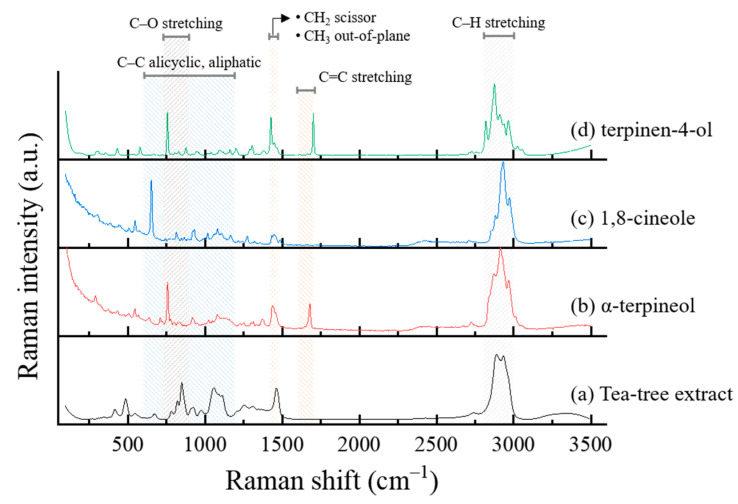
Raman spectra of (**a**) tea tree extract, (**b**) α-terpineol, (**c**) 1,8-cineole, and (**d**) terpinen-4-ol.

Figure 8a–d shows the Raman spectra of tea tree extract and its main constituents. The peaks between 2800 and 3000 cm^−^^1^ are ascribed to the C–H stretching vibrations. A peak at around 1650 cm^−1^ is assigned to the C=C stretching vibrations. The peaks arising from the vibrations of methylene (CH_2_) and/or alkyl (CH_3_) groups at around 1450 cm^−1^. The C–O stretching vibrations in the range of 750–900 cm^−1^ and the vibrations of the C–H group in the range of 600–1200 cm^−1^ can be identified [28,29,30].

**Figure 9:**
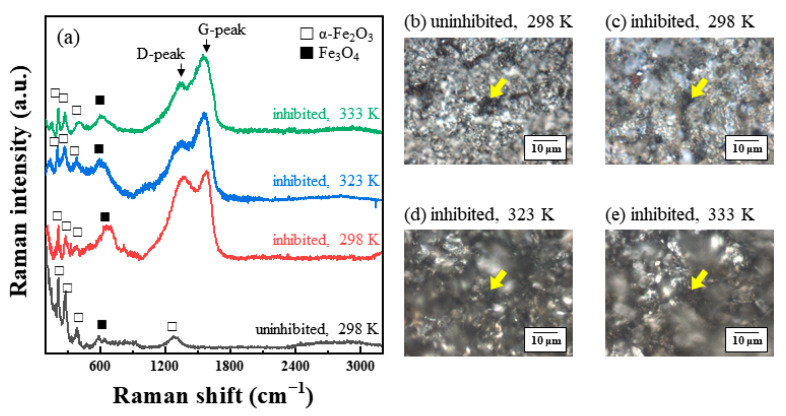
(**a**) Raman spectra analyzed from the surface of MS specimens after immersion for 24 h in 1 M HCl solution at 298, 323, and 333 K with 0.75 g/L of tea tree extract. (**b**–**e**) OM images showing the surface morphology of corroded MS specimens. The spots of Raman spectroscopic measurement are marked by arrows in OM images.

**Figure 10:**
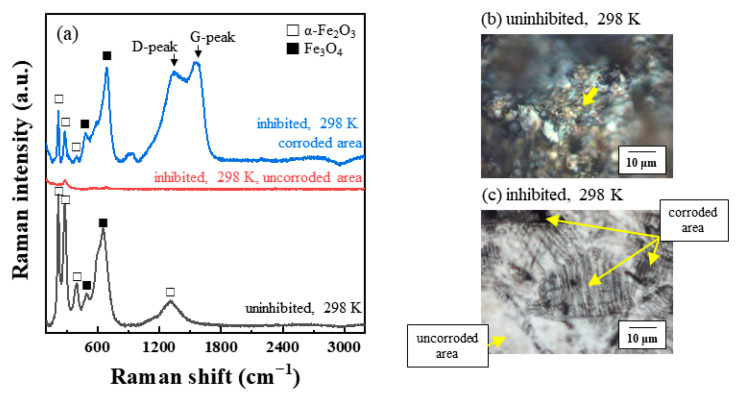
(**a**) Raman spectra analyzed from the surface of STS specimens after immersion for 168 h in 1 M HCl solution at 298 K with 0.75 g/L of tea tree extract. (**b**,**c**) OM images showing the surface morphology of corroded STS specimens. Spots of Raman spectroscopic measurement are marked by arrows in OM images.

**Figure 11:**
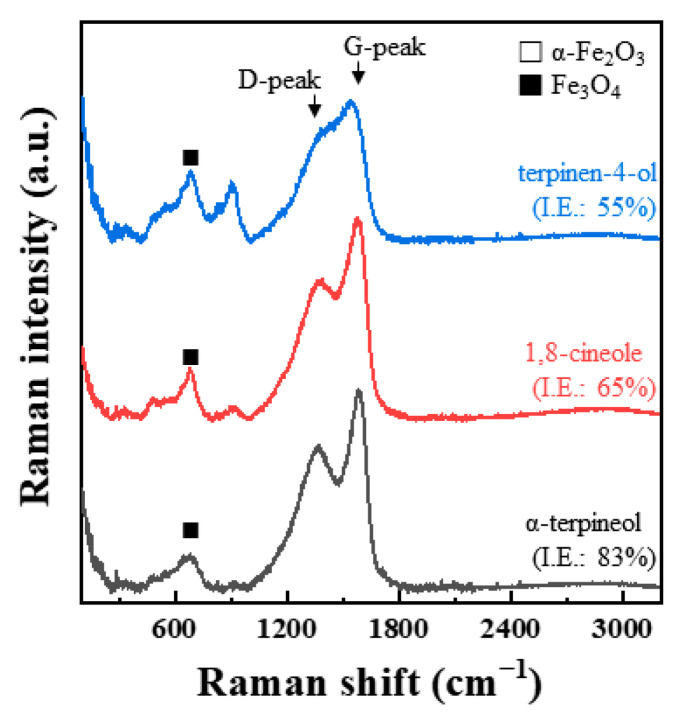
Raman spectra analyzed from the surface of STS specimens after immersion for 168 h in 1 M HCl solution at 298 K with α-terpineol, 1,8-cineole, and terpinen-4-ol of 0.75 g/L.

The Raman spectra and OM images were analyzed from the corroded surface of the MS and STS specimens (Figure 9, Figure 10 and Figure 11). The spots of Raman spectroscopic measurement are marked by arrows in the corresponding OM images. Additional Raman spectra were measured at different points, as shown in the Supporting Information (Figures S4–S6). Figure 9a compares the Raman spectra of MS specimens immersed in 1 M HCl solution at 298, 323, and 333 K for 24 h with or without 0.75 g/L of tea tree extracts. Two forms of iron oxides, α-Fe_2_O_3_ and Fe_3_O_4_, were identified as the corrosion products for all corroded MS specimens [31,32,33]. α-Fe_2_O_3_ and Fe_3_O_4_ exhibited the strongest signals at around 210 cm^−1^ and 692 cm^−1^, respectively [31,32]. Raman spectroscopic measurements of the STS specimens were also carried after immersion for 168 h in 1 M HCl solution at 298 K (Figure 10a). The results of the STS specimens on the iron oxide were similar to the results of the MS specimens.

In general, a G-peak at 1560 cm^−1^ and a D-peak at 1360 cm^−1^ have been observed for the various carbon materials such as metallocorroles [34,35,36], amorphous carbons [37,38], and graphitic materials [39,40]. Interestingly, G- and D-peaks were detected from our inhibition study. The corresponding G- and D-peaks were observed from the Raman spectra of the inhibited MS specimens. However, any significant peaks were not seen at 1560 or 1360 cm^−1^ in the case of the uninhibited specimen (Figure 9a). Similar to the results of the MS specimen, two peaks, G- and D-peaks, with significant intensities were obtained from the inhibited STS specimen (Figure 10a). Raman spectra of immersed STS specimens with α-terpineol, 1,8-cineole, and terpinen-4-ol are combined into one figure for easy comparison (Figure 11). G- and D-peaks were detected from all samples, and the most significant peaks were observed from the STS specimen inhibited by α-terpineol, 83% IE_WL_.

We assumed the presence of G- and D-peaks for the inhibited steel specimens indicates the adsorption of tea tree extract on the steel surface. Shifted or newly formed peaks in Raman spectra may be due to chemical interactions between the organic inhibitor and the steel surface by the adsorption. In addition, G- and D-peaks were observed only on the corroded area (Figure 10a,c). This result indicates that tea tree extract adsorbed only on the non-passivated area (i.e., the corrosive area), which coincides with previous studies of corrosion inhibition for stainless steel [41,42]. They explained that the adsorption of organic inhibitors occurred on a bare stainless-steel surface originating from the local breakdown of a passive layer. Therefore, we could conclude that tea tree extract was adsorbed through the interaction with the bare steel surface before attacking the corrosive ions. The appearance of G- and D-peaks is believed to be related to the adsorption of the organic inhibitor on the bare steel surface. Although the oxidation of Fe on the unprotective steel surface (i.e., dissolution of steel) occurred in 1 M HCl solution containing tea tree extract, further oxidation processes could be effectively retarded by this adsorption phenomenon.

**Figure 12:**
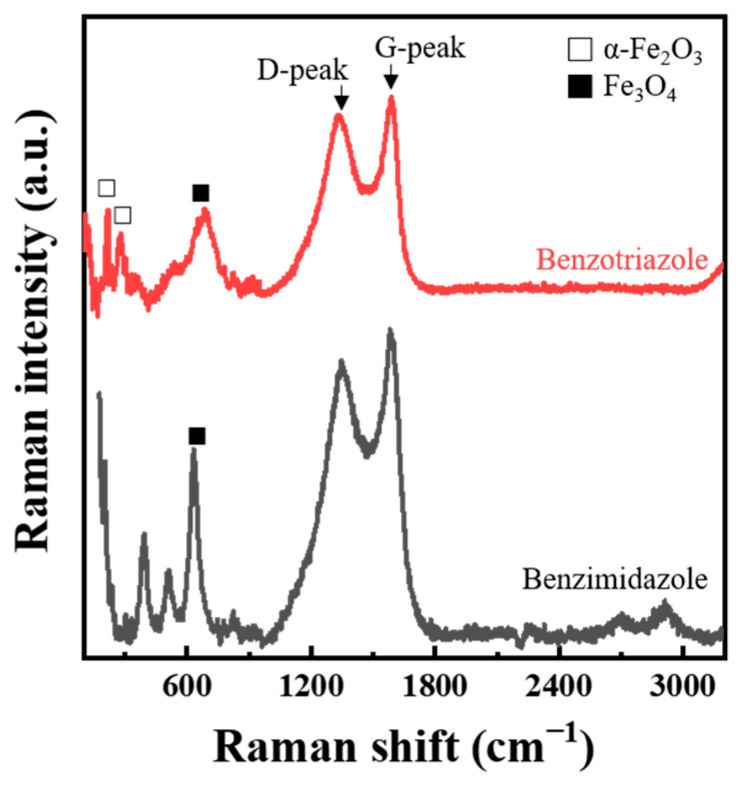
Raman spectra analyzed from the surface of STS specimens after immersion for 168 h in 1 M HCl solution at 298 K with benzimidazole and benzotriazole.

Our study regarding G- and D-peaks of Raman spectra is the first example in the field of corrosion inhibition as far as we searched. For a better understanding, well-studied organic corrosion inhibitors, benzimidazole [43,44] and benzotriazole [45,46], were also tested. The IE_WL_ of benzimidazole and benzotriazole on STS were 83% and 88%, which were obtained in the same manner as the weight loss test in Section 2.1. The surfaces of the inhibited STS specimens with benzimidazole and benzotriazole were analyzed by Raman spectroscopy (Figure 12 and Figure S7). Peaks related to α-Fe_2_O_3_ and Fe_3_O_4_ were detected between 200 and 900 cm^−1^. Interestingly, strong G- and D-peaks were also observed from the inhibition experiments with both organic inhibitors, and these results are similar to those of the study on tea tree extract. Therefore, we could conclude that the observation of G- and D-peaks is common in the corrosion inhibition test with organic inhibitors after immersion in acidic media.

**Figure 13:**
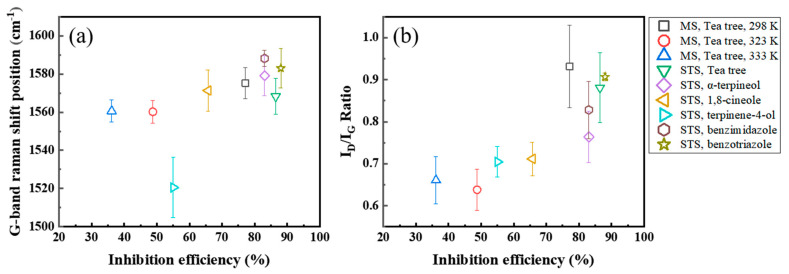
Behavior of (**a**) the G-peak position and (**b**) the I_D_/I_G_ intensity ratio with the IE_WL_ of organic inhibitors.

The intensity ratio of G- and D-peaks, I_D_/I_G_, and the G-peak position were analyzed based on the IE_WL_ (Figure 13) and are presented in Table 4. The G-peak shifted to higher frequencies in the case of a higher inhibition efficiency (Figure 13a). Even though the result of the STS specimen with terpinene-4-ol was out of the range, the others had a positive tendency of higher G- band frequencies with a higher IE_WL_. The I_D_/I_G_ intensity ratio had a positive correlation with the IE_WL_ of inhibitors (Figure 13b), and the I_D_/I_G_ intensity ratio increased as the IE_WL_ increased. Greater than 70% IE_WL_ of MS or STS specimens with organic inhibitors showed at least a 0.77 I_D_/I_G_ intensity ratio.

#### 3.4.3. Electrochemical Impedance Spectroscopy (EIS)

**Figure 14:**
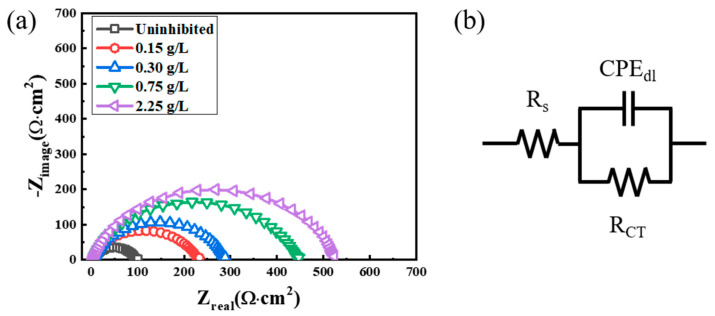
(**a**) Nyquist plots measured during the corrosion of MS specimens in 1 M HCl solution at 298 K containing different concentrations of tea tree extract; (**b**) equivalent circuit.

EIS provides useful information about the characteristics of the electric double layer. This layer is formed between the steel surface and the electrolyte. Thus, the adsorption of organic molecules on the steel surface can affect the impedance parameters of this layer [47]. Figure 14a shows Nyquist plots measured during the corrosion of the MS specimens in 1 M HCl solution at 298 K. The Nyquist plots in both the uninhibited and inhibited MS specimens exhibited one depressed semi-circle. One depressed semi-circle is a typical characteristic of steel electrodes with an inhomogeneous surface [48]. The equivalent circuit used in this study is shown in Figure 14b. To analyze the Nyquist plot of one semi-circle, Randle’s circuit consisting of solution resistance (R_S_), charge transfer resistance (R_CT_), and double-layer capacitance (C_dl_) is generally applied. However, for the Nyquist plot of one depressed semi-circle, the double-layer capacitance (C_dl_) is replaced with a constant phase element (CPE_dl_), as shown in the equivalent circuit of Figure 14b. As summarized in Table 5, electrochemical impedance parameters were analyzed by the proposed equivalent circuit model (Figure 14b). The C_dl_ is estimated using the CPE_dl_ according to Equation (5):

(5) $$C_{\mathrm{dl}}={\left({\mathrm{QR}}_{\mathrm{CT}}^{1-n}\right)}^{\frac{1}{n}},$$

where Q is the CPE_dl_ constant, and n is the CPE_dl_ exponent (0 ≤ n ≤ 1) related to the degree of surface inhomogeneity [49].

C_dl_ depends on a dielectric constant and thickness of electric double layer according to Equation (6):

(6) $$C_{\mathrm{dl}}=\frac{\varepsilon_{i}\varepsilon_{0}}{d},$$

where ε_0_ and ε_i_ are the local dielectric constant values of the electric double layer formed for the conditions in the absence and presence of the extract, respectively, and “d” means the thickness of the electric double layer [50].

C_dl_ was decreased, and R_CT_ was increased by the addition of tea tree extract in 1 M HCl solution. According to Equation (6), the decrease in C_dl_ values is attributed to an increase in d and/or a decrease in ε_i_. The organic constituents of tea tree extract may adsorb through displacement with the pre-adsorbed water molecules and corrosive ions on the steel surface. The adsorption of organic molecules on the steel surfaces may increase d and/or decrease ε_i_ [49]. The increase in R_CT_ values is because the adsorption layer acts as a protective physical barrier retarding the charge transfer on the steel surface. The IE_EIS_ was improved by up to about 80% with the higher concentration of extract, which is consistent with the weight loss test in Figure 1 and polarization measurement in Figure 4. These results of EIS confirm that tea tree extract is successfully adsorbed on the steel surface and effectively retards the steel corrosion.

### 3.5. Corrosion Inhibition Mechanism

**Figure 15:**
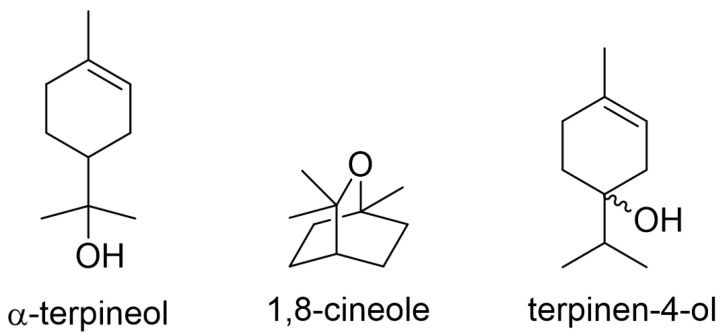
Main components of tea tree extract.

**Figure 16:**
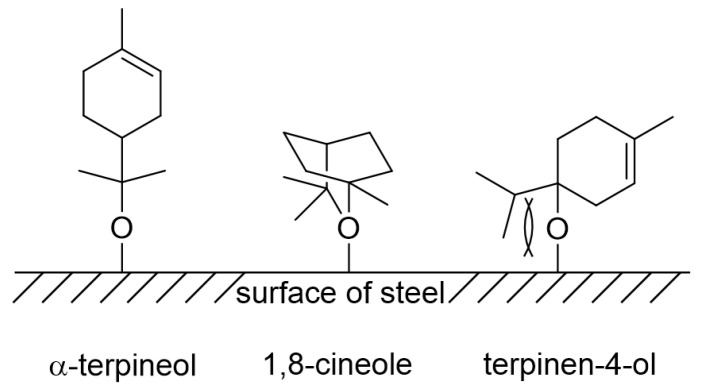
Adsorption of the main constituents on the surface of steel.

Organic compounds containing oxygen, nitrogen, sulfur, and phosphorus atoms were found to be effective inhibitors for corrosion because of their ability of adsorption on steel surfaces. In general, adsorption depends on the structures of organic compounds and the nature of functional groups. The main components of tea tree extract, α-terpineol, 1,8-cineole, and terpinen-4-ol, are organic compounds containing oxygen atom (Figure 15). Oxygen of a hydroxyl functional group in α-terpineol and terpinen-4-ol or the ether moiety in 1,8-cineole may effectively adsorb at the iron surface to make Fe-organic complexes by electron donor–acceptor interactions (Figure 16).

**Figure 17:**
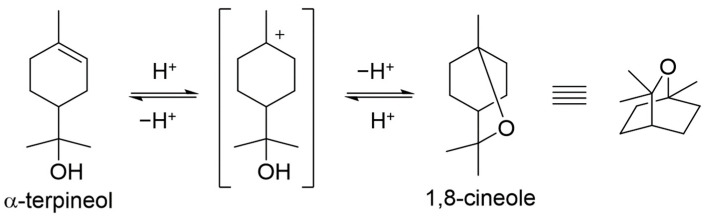
Isomerization of α-terpineol and 1,8-cineole.

Among three constituents, α-terpineol, 1,8-cineole, and terpinen-4-ol, α-terpineol act as the most effective inhibitors with 83% IE_WL_ (Figure 6). 1,8-Cineole shows 65% IE_WL_, and terpinen-4-ol has the lowest IE_WL_ of 55%. The hydroxyl group in α-terpineol adsorbs at the iron surface effectively to form a Fe-organic complex to inhibit corrosion by blocking the active site of the iron surface. The hydroxyl group could strongly adsorb to the steel surface by electron donor and electron acceptor (iron surface, Fe^2+^) interactions. The oxygen atom of ether in 1,8-cineole could also interact with Fe; however, this interaction is weaker than that of the hydroxyl group. Moreover, the steric bulkiness around the oxygen atom could avoid the efficient adsorption of 1,8-cineole to the steel surface. Even though 1,8-cineole is not an effective organic inhibitor due to the issues described above, 1,8-cineole has better corrosion efficiency than terpinen-4-ol. It might be explained by the isomerization of 1,8-cineole to α-terpineol in acidic media (Figure 17); therefore, some portion of 1,8-cineole exists as α-terpineol, which has good corrosion efficiency. In the case of terpinen-4-ol, the oxygen atom of the hydroxyl group is hard to interact with the surface of steel due to steric hindrance around the hydroxyl group. Therefore, the IE_WL_ is only 55% (Figure 6).

The corrosion inhibition mechanism of tea tree extract on MS and STS corrosion is proposed as follows:

- Chloride anions adsorb onto the positively charged steel surface in 1 M HCl solution by coloumbic attraction and cause steel dissolution [50,51].
- In the case of passivated STS, the adsorbed chloride anions cause local breakdown of the protective passive layers on the steel–solution interface, resulting in pitting corrosion.
- In the presence of tea tree extract, organic inhibitors containing oxygen adsorb to the corrosive areas, i.e., all surface areas for MS of uniform corrosion morphology (Figure 9a) and the local pits for STS of pitting corrosion morphology (Figure 10a). The pre-adsorbed chloride anions are replaced by organic inhibitors on the surface of the steel [50,51], and the organic-Fe complex layer is formed through electron donor–acceptor interactions with Fe. This layer effectively blocks the interaction between the steel and acid media and reduces the chance of the further oxidation of iron. The uniform corrosion of MS and pitting corrosion of STS in the 1 M HCl solution can effectively be inhibited by the protective organic-Fe complex layer.

## 4. Conclusions

Tea tree extract has been applied only in the cosmetic, food, and pharmaceutical industries. This study showed tea tree extract as a green promising corrosion inhibitor to substitute chemically synthesized compounds. Tea tree extract and its constituents α-terpineol, 1,8-cineole, and terpinen-4-ol exhibited an anticorrosive effect under 1 M HCl solution on uniform corrosion for MS and pitting corrosion for STS. The corrosion of MS and STS at 298 K was inhibited by up to 77% and 86%, respectively. LSCM observations showed that the addition of tea tree extract in 1 M HCl solution reduced the pitting area and depth for STS. The most effective constituent contributing to the inhibitory performance was found to be α-terpineol with an inhibition efficiency of 83%.

The anticorrosion mechanism of tea tree extract was revealed through surface characterization analysis using FTIR, Raman spectroscopy, and EIS. Organic-Fe complex layers were formed on the steel surface via electron donor and acceptor interactions in the presence of an oxygen atom of the hydroxyl group or ether of organic inhibitors. This layer retarded the corrosion reaction by blocking the interaction between the steel and acid media.

For Raman spectral analysis, the study regarding G- and D-peaks was firstly reported in the field of corrosion inhibition. The G- and D-peaks were detected from the Raman spectra of the inhibited specimens. The I_D_/I_G_ ratio and G- band frequencies had a positive relationship with the corrosion inhibition efficiency. Based on these empirical findings from the Raman spectroscopic results, a clearer adsorption and inhibition mechanism of organic materials on the steel will need to be studied further.

## Figures and Tables

**Table 1:** Chemical composition (wt%) of mild steel (MS) and 304 stainless steel (STS).

Type	C	Si	Mn	Cr	Ni	Cu	Al	Nb	P	S	Fe
MS	0.07	0.02	0.7	0.005	0.005	0.02	0.03	0.01	0.009	0.003	Bal.
STS	0.04	0.42	1.15	18.19	8.08	-	-	-	0.031	0.001	Bal.

**Table 2:** Operation specifications for GC–MS.

Gas Chromatograph (HP 6890, Hewlett-Packard Co., USA)			
Analytical column	HP-5MS (30 m × 0.25 mm × 0.25 μm)		
Inlet temperature	493 K		
Injection type	Split (5:1)		
Injection volume	1 μL		
Carrier gas	He (1 mL/min)		
Oven program	Temperature	Hold Time	Rate
	323 K	2 min	283 K/min
	523 K	3 min	
**Mass Spectrometer (HP 5973, Hewlett-Packard Co., USA)**			
Ionization	Electron ionization (EI), 70 eV		
Ion source temperature	203 K		
Quadrupole temperature	423 K		
MS transfer line temperature	553 K		
Mass range	SCAN mode (m/z 40–550)		
Solvent delay	3 min		

**Table 3:** Corrosion data obtained from the electrochemical corrosion tests in Figure 4.

Extract (g/L)	E_corr_ (V vs. SCE)	I_corr_ (A/cm^2^)	IE_PD_ (%)
Uninhibited	−0.492	1.79 × 10^−4^	
0.15	−0.484	0.78 × 10^−4^	56.5
0.30	−0.467	0.64 × 10^−4^	64.0
0.75	−0.461	0.44 × 10^−4^	75.6
2.25	−0.482	0.38 × 10^−4^	78.6

**Table 4:** The intensity ratio of the D-peak to G-peak (I_D_/I_G_) and the G-peak position (cm^−1^) evaluated in Figure 9, Figure 10, Figure 11 and Figure 12.

Inhibitor	Specimen	Temperature (K)	IE_WL_ (%)	I_D_/I_G_	G-Peak Position (cm^−1^)
tea tree extract	MS	298	77	0.92	1576
		323	48	0.67	1560
		333	36	0.66	1558
tea tree extract	STS	298	86	0.88	1568
α-terpineol			83	0.77	1579
1,8-cineole			65	0.71	1571
terpinen-4-ol			55	0.7	1520
benzimidazole			83	0.83	1588
benzotriazole			88	0.91	1583

**Table 5:** Electrochemical impedance parameters obtained from Figure 14a.

Extract (g/L)	R_S_ (Ω·cm^2^)	R_CT_ (Ω·cm^2^)	CPE_dl_ (μs/Ω·cm^2^)	n	C_dl_ (μF/cm^2^)	IE_EIS_ (%)
Uninhibited	4.9	91	128	0.8281	51.8	
0.15	2.5	224	110	0.8315	52.0	59.3
0.30	4.2	280	106	0.8395	54.1	67.4
0.75	4.6	432	70	0.8447	36.8	78.9
2.25	4.2	515	65	0.8523	36.1	82.3

## Data Availability

Not applicable.

## Supplementary Materials

The following are available online at https://www.mdpi.com/article/10.3390/ma14175016/s1, Figure S1: Raman spectra analyzed with 1, 4, 7, and 10 mW laser power from (a,b) the extract itself and (c,d) the corroded surface of MS specimen without the extract. Thermal degradation with 10 mW laser power was checked via the repetitive analysis at one spot, as shown in (b,d), Figure S2a–g: LSCM images showing the surface morphology of corroded 304 STS specimens after immersion in 1 M HCl solution at 298 K without tea tree extract, Figure S3a–g: LSCM images showing the surface morphology of corroded 304 STS specimens after immersion in 1 M HCl solution at 298 K with 0.75 g/L of tea tree extract, Figure S4: Raman spectra analyzed from the surface of MS specimens after immersion for 24 h in 1 M HCl solution at (a,b) 298, (c) 323, and (d) 333 K with 0.75 g/L of tea tree extract, Figure S5: Raman spectra analyzed from the surface of STS specimens after immersion for 168 hours in 1 M HCl solution at 298 K (a) without and (b) with tea tree extract of 0.75 g/L of tea tree extract, Figure S6: Raman spectra analyzed from the surface of STS specimens after immersion for 168 hours in 1 M HCl solution at 298 K with 0.75 g/L of (a) α-terpineol, (b) 1,8-cineole, and (c) terpinen-4-ol, Figure S7: Raman spectra analyzed from the surface of STS specimens after immersion for 168 h in 1 M HCl solution at 298 K with (a) benzimidazole and (b) benzotriazole.
