# Supplementary material for: Corrosion Inhibition of Mild Steel and 304 Stainless Steel in 1 M Hydrochloric Acid Solution by Tea Tree Extract and Its Main Constituents
Source: Materials (Basel). 2021 Sep 2;14(17):5016. doi: 10.3390/ma14175016 (PMC8433979; doi:10.3390/ma14175016)
Supplement: Supplementary file 1 [file materials-14-05016-s001.zip › materials-1334500-SI.pdf]

Corrosion Inhibition of Mild Steel and 304 Stainless Steel in 1 M Hydrochloric Acid Solution by Tea Tree Extract and Its Main Constituents

Jae-Yeon Kim [1,2,†], **InJi Shin** [3,*] and **Jai-Won Byeon** [4,*]

1 Program of Material Science and Engineering, Convergence Institute of Biomedical Engineering and Biomaterials, Seoul National University of Science and Technology, Seoul 01811, Korea
2 Department of Mining and Geological Engineering, University of Arizona, Tucson, AZ 85721, USA
3 Department of Fine Chemistry, Seoul National University of Science and Technology, Seoul 01811, Korea
4 Department of Materials Science and Engineering, Seoul National University of Science and Technology, Seoul 01811, Korea
* Correspondence: inji@seoultech.ac.kr (I.S.); @seoultech.ac.kr (J.-W.B.); Tel.: +82-2-970-6690 (I.S.); +82-2-970-6634
† Present address: Department of Mining and Geological Engineering, University of Arizona, Tucson, AZ 85721, USA.

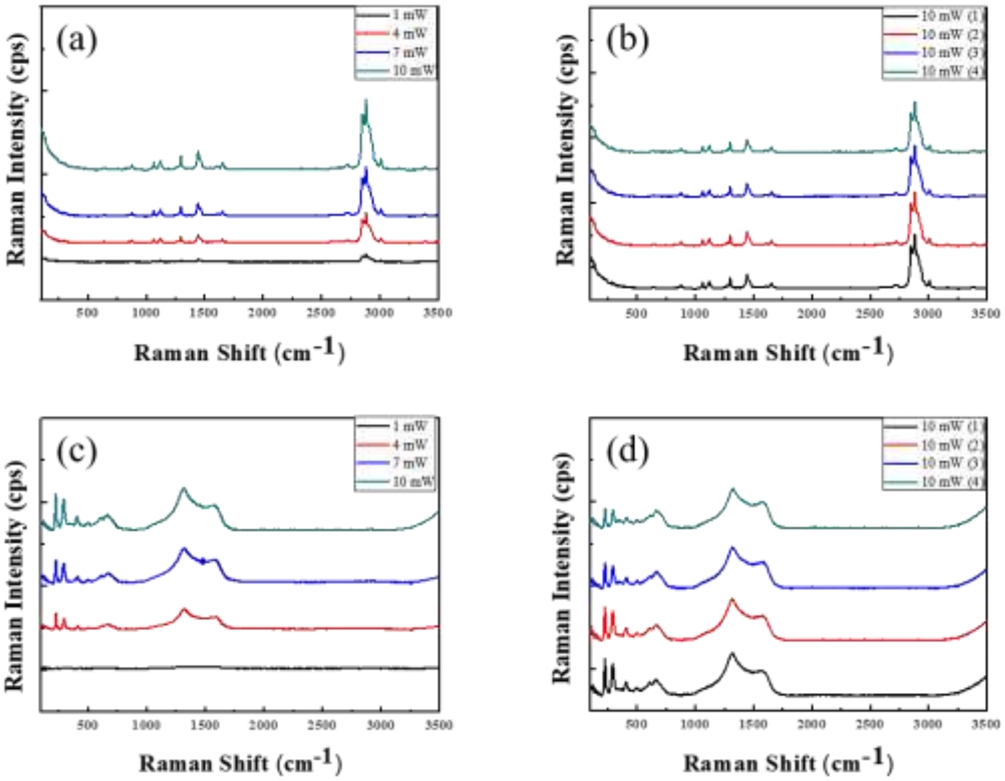

Figure S1. Raman spectra analyzed with 1, 4, 7, and 10 mW laser power from (**a**,**b**) the extract itself and (**c**,**d**) the corroded surface of the MS specimen without the extract. Thermal degradation with 10 mW laser power was checked via repetitive analysis at one spot, as shown in (**b**,**d**).

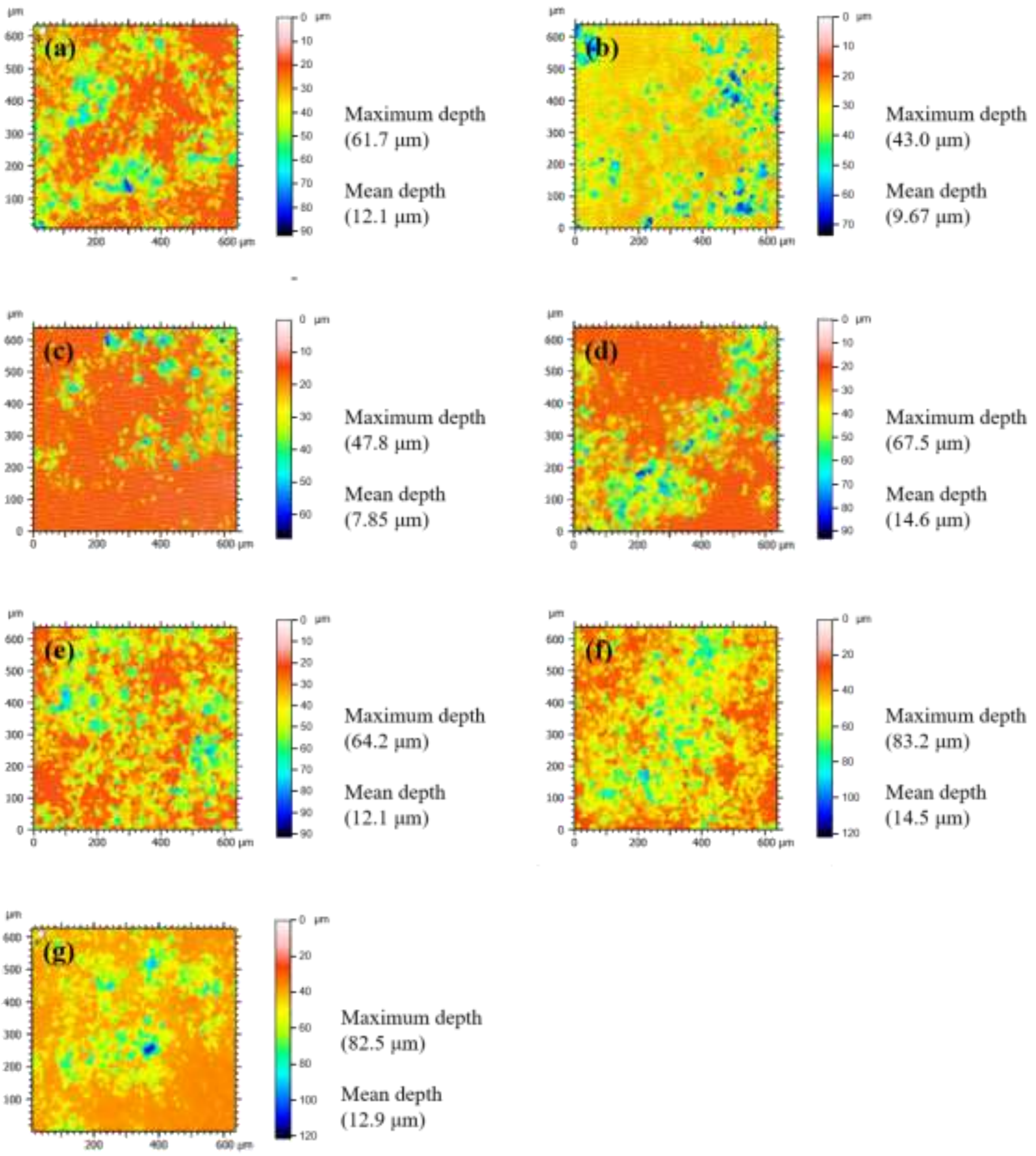

Figure S2. (**a–g**) LSCM images showing the surface morphology of corroded 304 STS specimens after immersion in 1M HCl solution at 298 K without tea tree extract.

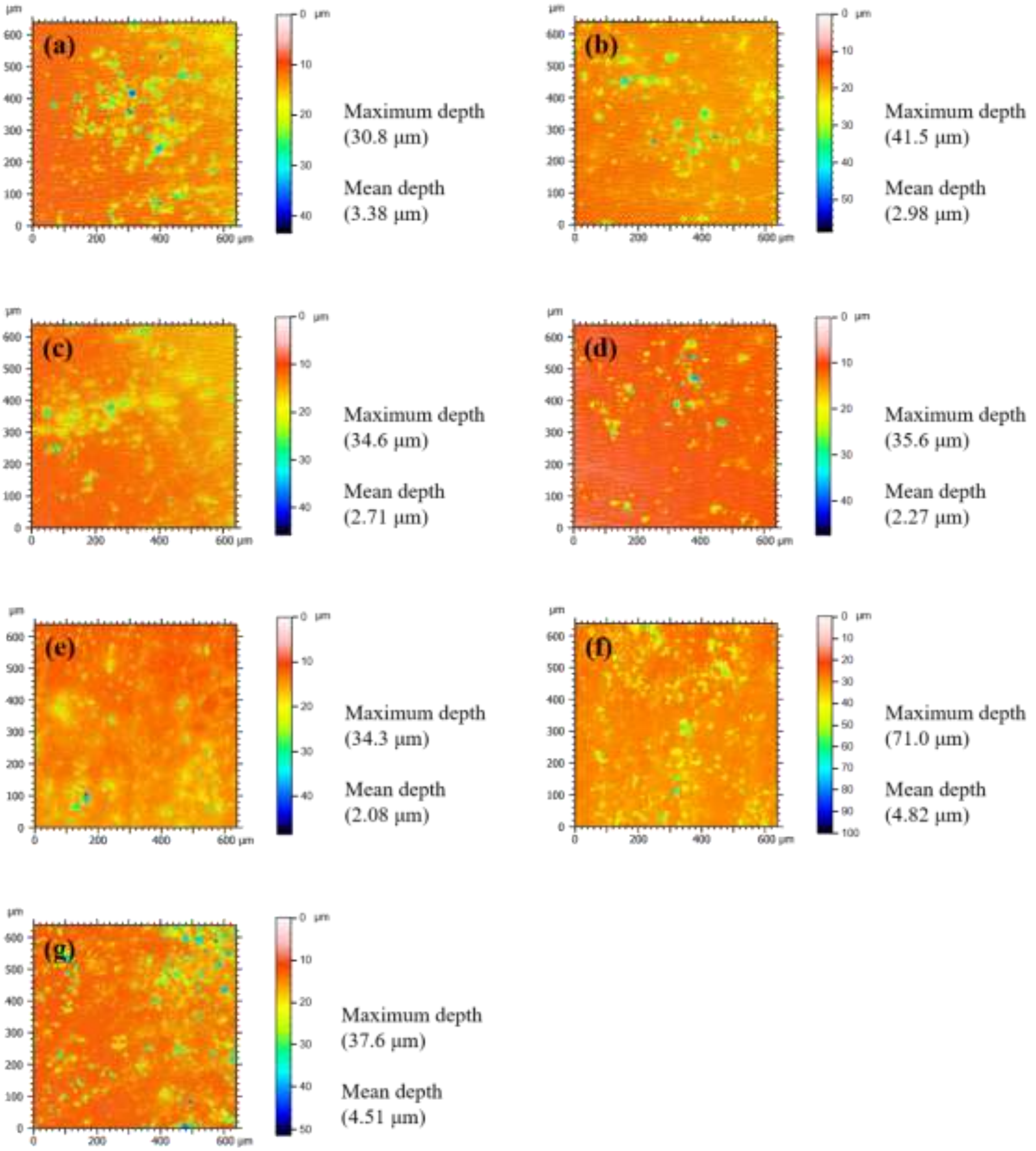

Figure S3. (**a**–**g**) LSCM images showing the surface morphology of corroded 304 STS specimens after immersion in 1M HCl solution at 298 K with 0.75 g/L of tea tree extract.

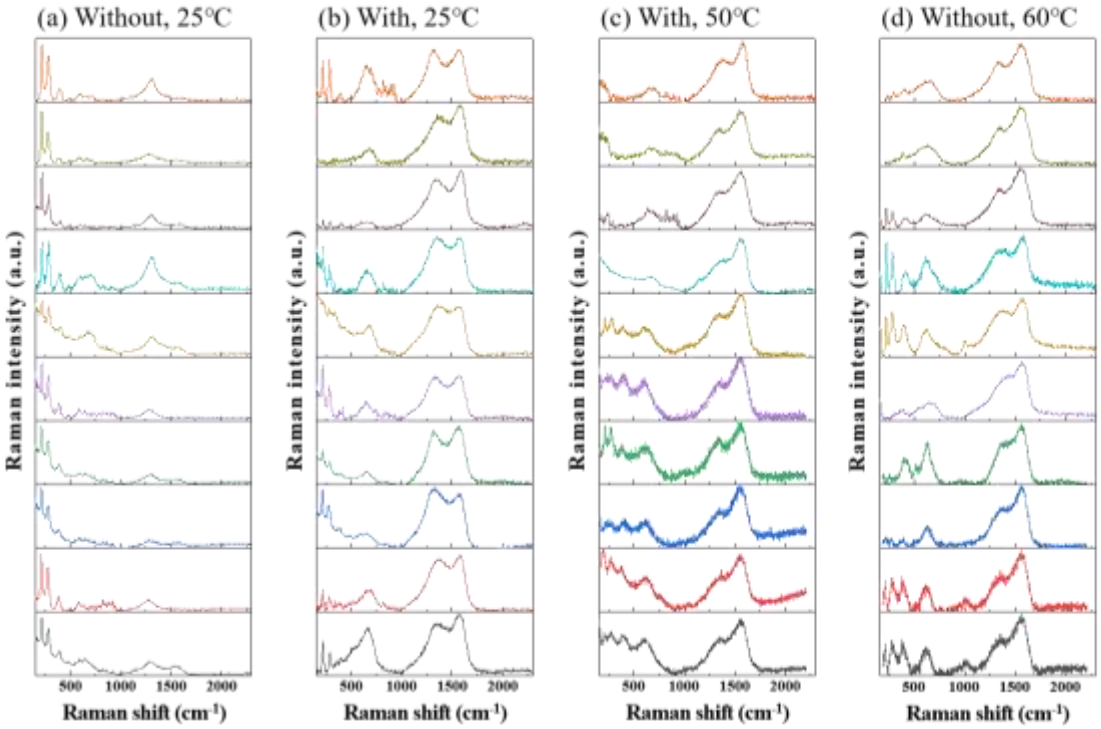

Figure S4. Raman spectra analyzed from the surface of MS specimens after immersion for 24 h in 1 M HCl solution at (**a**,**b**) 298, (**c**) 323 and (**d**) 333 K with 0.75 g/L of tea tree extract.

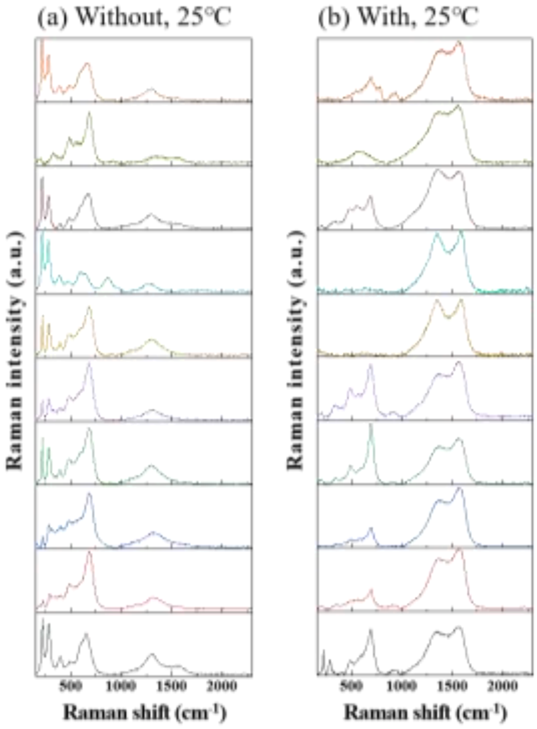

Figure S5. Raman spectra analyzed from the surface of STS specimens after immersion for 168 h in 1 M HCl solution at 298 K (**a**) without and (**b**) with 0.75 g/L of tea tree extract.

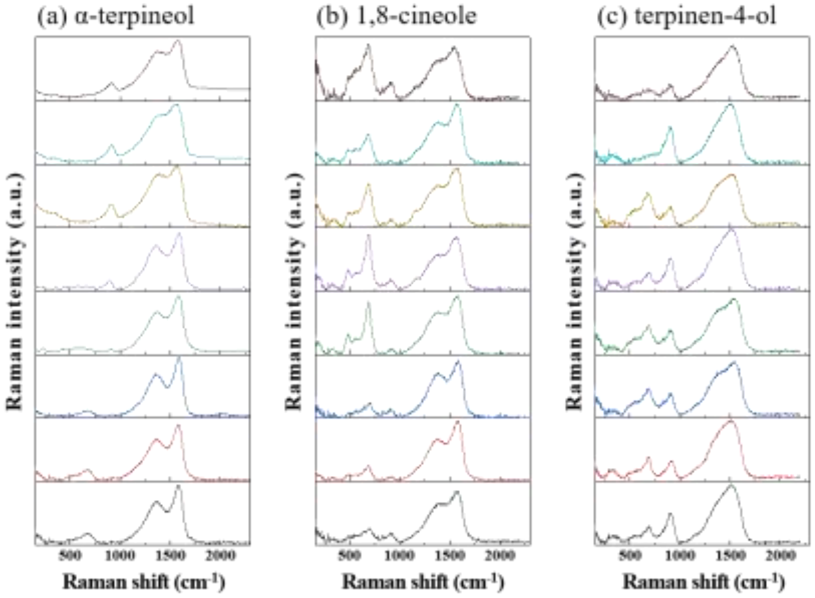

Figure S6. Raman spectra analyzed from the surface of STS specimens after immersion for 168 h in 1 M HCl solution at 298 K with 0.75 g/L of (**a**) α-terpineol, (**b**) 1,8-cineole and (**c**) terpinen-4-ol.

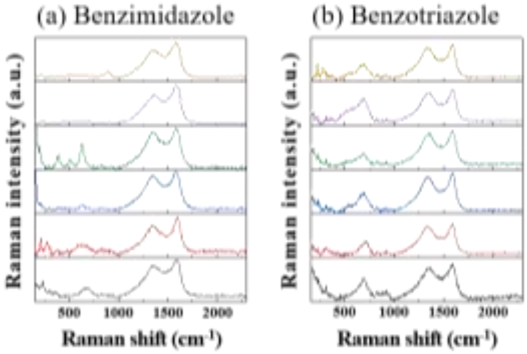

Figure S7. Raman spectra analyzed from the surface of STS specimens after immersion for 168 h in 1 M HCl solution at 298 K with (**a**) benzimidazole and (**b**) benzotriazole.
